# Implications of current therapeutic restrictions for primaquine and tafenoquine in the radical cure of vivax malaria

**DOI:** 10.1371/journal.pntd.0006440

**Published:** 2018-04-20

**Authors:** James Watson, Walter R. J. Taylor, Germana Bancone, Cindy S. Chu, Podjanee Jittamala, Nicholas J. White

**Affiliations:** 1 Mahidol Oxford Research Unit, Faculty of Tropical Medicine, Mahidol University, Bangkok, Thailand; 2 Centre for Tropical Medicine and Global Health, Nuffield Department of Medicine, Oxford University, Oxford, United kingdom; 3 Shoklo Malaria Research Unit, Faculty of Tropical Medicine, Mahidol University, Mae Sot, Thailand; 4 Department of Clinical Tropical Medicine, Faculty of Tropical Medicine, Mahidol University, Bangkok, Thailand; Johns Hopkins Bloomberg School of Public Health, UNITED STATES

## Abstract

**Background:**

The 8-aminoquinoline antimalarials, the only drugs which prevent relapse of vivax and ovale malaria (radical cure), cause dose-dependent oxidant haemolysis in individuals with glucose-6-phosphate dehydrogenase (G6PD) deficiency. Patients with <30% and <70% of normal G6PD activity are not given standard regimens of primaquine and tafenoquine, respectively. Both drugs are currently considered contraindicated in pregnant and lactating women.

**Methods:**

Quantitative G6PD enzyme activity data from 5198 individuals were used to estimate the proportions of heterozygous females who would be ineligible for treatment at the 30% and 70% activity thresholds, and the relationship with the severity of the deficiency. This was used to construct a simple model relating allele frequency in males to the potential population coverage of tafenoquine and primaquine under current prescribing restrictions.

**Findings:**

Independent of G6PD deficiency, the current pregnancy and lactation restrictions will exclude ~13% of females from radical cure treatment. This could be reduced to ~4% if 8-aminoquinolines can be prescribed to women breast-feeding infants older than 1 month. At a 30% activity threshold, approximately 8–19% of G6PD heterozygous women are ineligible for primaquine treatment; at a 70% threshold, 50–70% of heterozygous women and approximately 5% of G6PD wild type individuals are ineligible for tafenoquine treatment. Thus, overall in areas where the G6PDd allele frequency is >10% more than 15% of men and more than 25% of women would be unable to receive tafenoquine. In vivax malaria infected patients these proportions will be lowered by any protective effect against *P*. *vivax* conferred by G6PD deficiency.

**Conclusion:**

If tafenoquine is deployed for radical cure, primaquine will still be needed to obtain high population coverage. Better radical cure antimalarial regimens are needed.

## Author summary

More than half of the malaria outside of Sub-Saharan Africa is caused by the parasite *Plasmodium vivax* which is characterised by multiple relapses of malaria from parasites which persist in the liver. The only drugs which prevent these relapses (radical cure) are the 8-aminoquinolines primaquine and tafenoquine, and they both cause haemolytic anaemia in G6PD deficiency, the most common enzymopathy of man. Neither can currently be prescribed in pregnancy or lactation. Tafenoquine is given as a single dose regimen and is a significant advance over primaquine (recommended as a 14 day regimen). However, a greater number of individuals, mostly females, will be ineligible for tafenoquine treatment due to a tighter restriction on the minimum G6PD enzyme activity considered safe for use of the drug. Using enzyme activity data from over 5000 individuals, we estimate the proportions ineligible due to G6PD deficiency as a function of the deficient allele prevalence. Adding this to simple estimates of pregnancy and lactation, we estimate the proportions of populations who cannot receive either tafenoquine or primaquine radical cure. For the elimination of vivax malaria in areas with a high prevalence of G6PD deficiency, then if tafenoquine is deployed primaquine will still be needed, so better regimens should be developed.

## Introduction

*Plasmodium vivax* is an important cause of malaria outside Sub-Saharan Africa. The WHO estimates that *P*. *vivax* comprises 41% of the malaria burden outside of Africa. This translates into 6–11 million cases/year with an estimated 1800–4900 deaths. India, Indonesia and Pakistan account for just over 80% of the global vivax malaria case burden [1]. Relapse frequencies vary by geographical region. South East Asia and Oceania have the highest incidence with relapse rates exceeding 50% [2]. In this context relapse from liver hypnozoites is the main cause of *P*. *vivax* malaria illness and asymptomatic carriage [3].

The only currently available treatment to eliminate liver hypnozoites and thus prevent future relapses (‘radical cure’) of vivax or ovale malaria is primaquine, a rapidly eliminated 8-aminoquinoline. Radical curative efficacy depends on the total dose administered [4]. Treatment courses of 14 days are recommended by the WHO but the effectiveness of unsupervised primaquine is often poor [5,6]. Primaquine has one major adverse effect–it causes dose related acute haemolytic anaemia (AHA) in individuals with glucose-6-phosphate dehydrogenase deficiency (G6PDd) [7].

G6PDd is a common inherited X-linked red blood cell disorder prevalent in tropical and subtropical regions, where in some ethnic groups 35% of males are G6PDd [8]. Males are either deficient (hemizygotes) or normal, whereas females can be fully deficient (homozygotes), partially deficient (heterozygotes) or normal. Due to random X-inactivation (Lyonisation), heterozygous females have two red blood cell populations, one with normal G6PD enzyme activity and the other with reduced activity. On average heterozygote females have half the red cell enzyme activity of normal individuals [9]. However, because X-inactivation occurs early in embryogenesis, there is significant variation between individual heterozygous females in the ratio of deficient to normal red cells.

Standard radical cure regimens of daily primaquine (0.25 or 0.5 mg/kg/day x 14d) are not given to patients who test as G6PDd with currently available qualitative rapid diagnostic tests (RDTs). These tests identify subjects with < 30% of normal activity [10–12] and so detect all male hemizygotes and female homozygotes but only some heterozygous females [13]. The majority of heterozygous females have G6PD enzyme activities above 30% but they may still experience AHA when given daily primaquine or single dose tafenoquine [14–16]. Data in this vulnerable group are limited.

The WHO currently recommends eight weekly primaquine [0.75 mg/kg (45 mg in adults)] doses for those with mild G6PD deficiency variants but its safety is uncertain in the more severe G6PDd variants such as the Mediterranean variant in the Middle East and west Asia, and the variants (e.g. Mahidol, Viangchan, Vanua Lava, Canton) prevalent in South East Asia and Oceania [17]. Weekly primaquine in Cambodia, where G6PD Viangchan predominates [11], resulted in one of 18 vivax infected G6PDd patients requiring a blood transfusion [18]. Accordingly, the WHO recommends a careful risk benefit assessment and medical supervision if weekly primaquine is given.

Tafenoquine is a slowly eliminated primaquine analogue (half life ~14d) that will soon be introduced as a single dose regimen to provide radical cure. Tafenoquine also causes dose dependent AHA in G6PDd individuals [16], but because its slow elimination provides a protracted oxidant effect, its use will be restricted to individuals whose G6PD enzyme activity is > 70% of normal. For tafenoquine, this will require the use of a G6PD test that can quantify the G6PD activity, posing a significant challenge for malaria control programmes.

Both primaquine and tafenoquine are contraindicated in pregnancy and during lactation for fear of causing AHA in the foetus or in a G6PDd breast-fed infant. However, recent work has shown that concentrations of primaquine in breast milk are very low and likely to be safe for G6PDd infants outside the neonatal period [19]. Current dosing restrictions curtail the use of primaquine and tafenoquine [12]. We examined the effect of these restrictions on the potential coverage of radical cure with primaquine today and tafenoquine in the future.

## Methods

### Ethics statement

All data used in this analysis were from trials with ethical approval where all subjects gave fully informed consent.

### Genetics

G6PDd is X-linked. The polymorphic variants prevalent in areas where malaria is or was prevalent confer varying levels of enzyme deficiency. We assume that the population distribution of the polymorphic G6PDd genotypes conforms to the Hardy-Weinberg equilibrium [20]. Hereafter, `allele frequency’ refers to the allele frequency in hemizygous males. In the studies (all studies except for [10,21,22]) where genotype data were not available, the expected number of homo- and heterozygous females was calculated from the Hardy-Weinberg proportions with deficient allele frequency estimated from the sampled male population.

### G6PD activity data and distribution of enzyme activities in heterozygous females

There are few large data sets of G6PD quantitative activities. Raw and meta-data were collected from nine recent studies (2013–2017) that reported quantitative measurements of G6PD activity. Three of these datasets were from studies in malaria patients (*P*. *vivax* and *P*. *falciparum*); four were in healthy volunteers; one was in pregnant women; and one was from a mass primaquine treatment study [10,13,21–27]. These nine studies represent a convenience sample of G6PD activity data.

Raw data were available or made available for seven of these studies and for the other two studies, the corresponding authors kindly provided the meta-data. The full extracted meta-data are provided in the supplementary materials. The methods are calibrated to the adjusted population median activity [28].

Total G6PD enzyme activity in heterozygous females is assumed to be normally distributed (data based assumption from [22]). The mean and variance of this distribution is expected to depend on the severity of the deficiency conferred by the hemizygous/homozygous genotypes. As the majority of studies did not have genotype corrected data, a Bayesian hierarchical model was used to estimate the quantiles of the distributions of enzyme activity corresponding 30 and 70% of the population median as a function of two separate categories of severity using a Bayesian beta-binomial model. This categorisation allows for partial correction of the over-dispersion in the data. We defined two categories, which roughly separate out the severity of the deficiencies as follows: Category 1: A- and Mahidol; Category 2: Viangchan, Orissa, and Vanua Lava. These categories were defined by calculating the ratio of the median activity in hemizygous deficient males over the median adjusted normal activity. This visually clustered the genotypes into these two categories. Note that these categories do not correspond to the WHO categories [29].

The Bayesian beta-binomial model was fitted in R using *stan* [30]. See supplementary materials for full model specification and code.

To estimate the distributions of G6PD activities in hemizygous males and homozygous females (theoretically identical) we pooled all activity data from G6PD normal males (classified by phenotype) and G6PD normal females (classified by genotype to avoid bias from heterozygotes). To adjust for inter-study variability, each G6PD activity was then scaled by $\frac{10}{study\;median\;activity}$ so that the pooled data had a median of 10 (arbitrary value).

### Current drug contraindications

Currently, neither primaquine nor tafenoquine can be given during pregnancy or breast-feeding and are not recommended in children < 6 months. The incidence of vivax malaria is usually low in young infants so they were excluded from the calculations.

In our main scenario, we assume that the average woman of reproductive age (15–40 y) has three children who survive at least two years and that each child is breast-fed for two years (the minimum recommended by WHO [31]). Fertility rates in Indonesia, Pakistan and India (which comprise more than 80% of annual cases) range from 2.4–3.4 (data.worldbank.org/indicator/SP.DYN.TFRT.IN/). This totals a period of 8.25 years during which women on average cannot take radical curative regimens.

We assume that in countries where *P*. *vivax* is endemic, women of reproductive age comprise 40% of the total female population (this corresponds to current Asian populations, see www.populationpyramid.net/asia/2016/).

### Calculations based on two G6PD test thresholds– 30 and 70%

For both tested thresholds, we have assumed that in a given population the male to female ratio is 1:1 and that the G6PDd allele frequency in hemizygous males can vary between 0 and 25%. For primaquine dosing, qualitative G6PD tests with thresholds of 30% are in use currently. Quantitative point-of-care tests are still being developed. In these calculations, we have assumed a suitable quantitative test is available (i.e. detects accurately at least 70% of normal activity).

For males, it is assumed that the qualitative test has 100% specificity and sensitivity. For the quantitative test with a 70% of population median threshold all deficient males are correctly identified and 5% of normal males are misclassified as deficient (see Results). Thus, the proportion of males who cannot receive either radical cure is equal to the background allele frequency (*q*) for primaquine, and slightly higher for tafenoquine (0.05 + 0.95q).

The proportions of females who cannot receive radical cure (*X_PQ_* for primaquine & *X_TQ_* for tafenoquine) under current prescribing restrictions were calculated as follows:

$$X_{PQ}=\Delta_{def}^{PQ}+0.4\times \frac{PBF}{25}-\left(0.4\times \frac{PBF}{25}\right)\Delta_{def}^{PQ},$$

$$X_{TQ}=\Delta_{def}^{TQ}+0.4\times \frac{PBF}{25}-\left(0.4\times \frac{PBF}{25}\right)\Delta_{def}^{TQ}.$$

Where $\Delta_{def}^{PQ}$ and $\Delta_{def}^{TQ}$ are the proportions of females who are classified as G6PD deficient at enzyme activity thresholds of 30 and 70% of the population median:

$$\Delta_{def}^{PQ}=q^{2}+2pqQ_{30\%},$$

$$\Delta_{def}^{TQ}=q^{2}+2pqQ_{70\%}+0.05(1-q).$$

*q*: G6PDd allele frequency; *p = 1-q*; *Q*_*30%*_ *& Q*_*70%*_: proportions of heterozygous females classified as G6PD deficient under a 30% & 70% cut-off, respectively; 0.4: the proportion of women between 15–40 years of age; PBF: mean number of years during which 8-aminoquinolines are restricted due to pregnancy or breast-feeding (this is a function of the fertility rate) and 25 is the number of years of reproductive age; 0.05: proportion of wild type (WT) homozygous females misclassified as deficient.

### Accession numbers

Extracted meta-data from studies included in analysis are available in the supplementary materials. Publicly available datasets used were:

https://doi.org/10.1371/journal.pone.0116143.s001

https://doi.org/10.1371/journal.pone.0169930.s002

https://doi.org/10.1371/journal.pone.0151898.s003.

## Results

### Quantitative G6PD activity data

Seven of the nine studies were in South East Asian populations (dominant G6PDd genotypes: Viangchan, Mahidol and Vanua Lava); one study was from Bangladesh (likely dominant genotypes Orissa, Kalyan-Kerala [32] and Mahidol); and one study was in African Americans (dominant genotype: A-) (Table 1).

**Table 1:** Meta-data from 9 studies which recorded quantitative G6PD activities. Population median values are adjusted as per the method of Domingo et al (2013). The full extracted table of data is provided in the supplementary materials.

Study No.	First Author and reference	Country	Year	Population	Allele frequency (%)	Females	Males	Heterozygotes < 30%	Heterozygotes < 70%	WT < 70%	Genotyping
1	Roca-Feltrer[13]	Cambodia	2014	Healthy volunteers	13	487	451	6	73	15	No
2	Ley[24]	Bangladesh	2017	Malaria patients (P.v. & P.f.)	12.7	598	401	22.8	85	1	No
3	Khim[23]	Cambodia	2013	Malaria patients (P.v.)	15	478	425	7	NA	20	No
4	LaRue[10]	USA: African Americans	2014	Healthy volunteers	14.9	107	107	0	43	1	Yes
5	Bancone[21]	Thailand	2016	Pregnant women	15	325	0	6	61	2.6	Yes
6	Oo[25]	Myanmar	2016	Healthy volunteers	11.1	476	524	4	47	1.7	No
7	Satyagraha[26]	Indonesia	2016	Healthy volunteers	9.2	260	350	29	62	3.4	No
8	Bancone[22]	Thailand	2016	MDA	15.6	69	39	7.8	NA	NA	Yes
9	Lin[27]	Cambodia	2017	Malaria patients (P.f.)	7	3	98	NA	NA	14.3	No

MDA: mass drug administration; P.f.: Plasmodium falciparum; P.v.: Plasmodium vivax; WT: wild type.

These studies recorded quantitative enzyme data for a total of 2803 females and 2395 males. Estimated G6PDd allele frequencies varied from 7–15%. The estimated proportions of heterozygous females with enzyme activity < 30% of the population median varied considerably from 0% (A- variant: estimated 0 out of ~27 expected heterozygotes) to 29% (Vanua Lava, exact numbers not reported). The estimated proportions of heterozygous females with activity < 70% of the population median also varied considerably from 43% (A- variant: estimated ~12 out of ~27 expected heterozygotes) to 85% (Orissa/Mahidol are likely variants: estimated ~113 out of ~133 expected heterozygotes).

**Fig 1:**
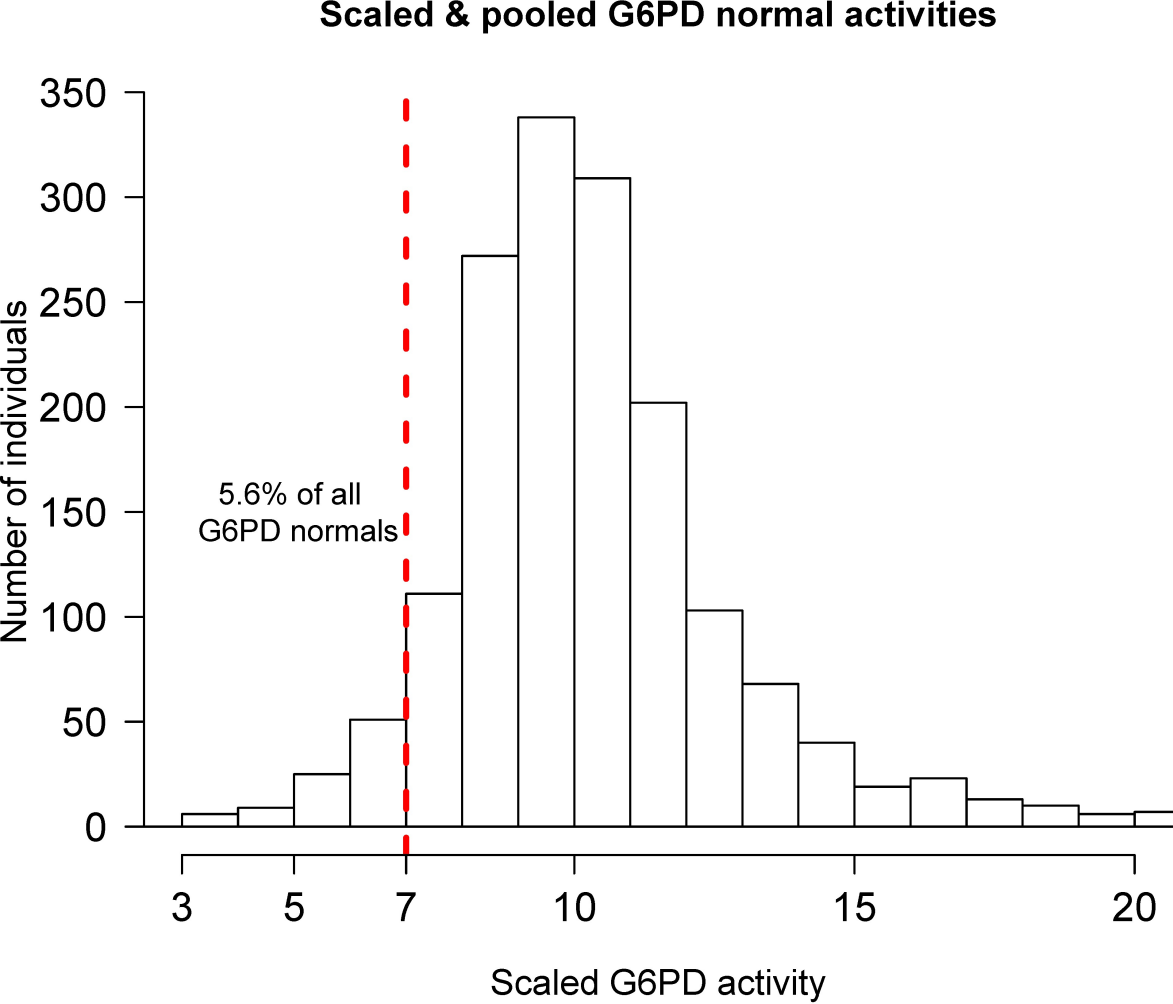
Distribution of G6PD activities in wild type males (determined from phenotype) and wild type females (only those determined from genotype to avoid bias from heterozygotes). Each activity has been scaled by 10/(Study median activity) to obtain a global median of 10. The units are relative to the corresponding study median. The 70% of population median threshold is shown by the vertical dashed red line.

Pooled individual enzyme activity data on G6PD WT males (classified by phenotype) and G6PD WT females (only those classified by genotype) were used to estimate the proportion of all G6PD WT individuals who would have G6PD enzyme activity below a 70% threshold. There was substantial variation in this proportion across studies, with estimates varying from 1–20% in males and 2.6% in females, with a mean estimate of 5.6% (Fig 1).

### Effect of G6PDd genotype on the distribution of activities in heterozygous females

We estimate that between 8% (African A- and Mahidol variants, 90% credible interval (C.I.): 2–19%), and 19% (Orissa, Viangchan and Vanua Lava variants, 90% C.I.: 9–36%) of heterozygous females test as G6PD deficient at a 30% threshold. The same model estimates that between 50% (African A- and Mahidol variants, 90% C.I.: 30–70) and 71% (Orissa, Viangchan and Vanua Lava variants, 90% C.I.: 51–86) of heterozygous females test as G6PD deficient at a 70% threshold. Both models conclude that an increasing level of G6PDd severity is associated with lower mean enzyme activities in heterozygotes [33,34].

To simplify results, all following calculations assume that 10 and 70% of heterozygous females classify as deficient at 30 and 70% thresholds, respectively.

### Implications of current prescribing restrictions

**Fig 2:**
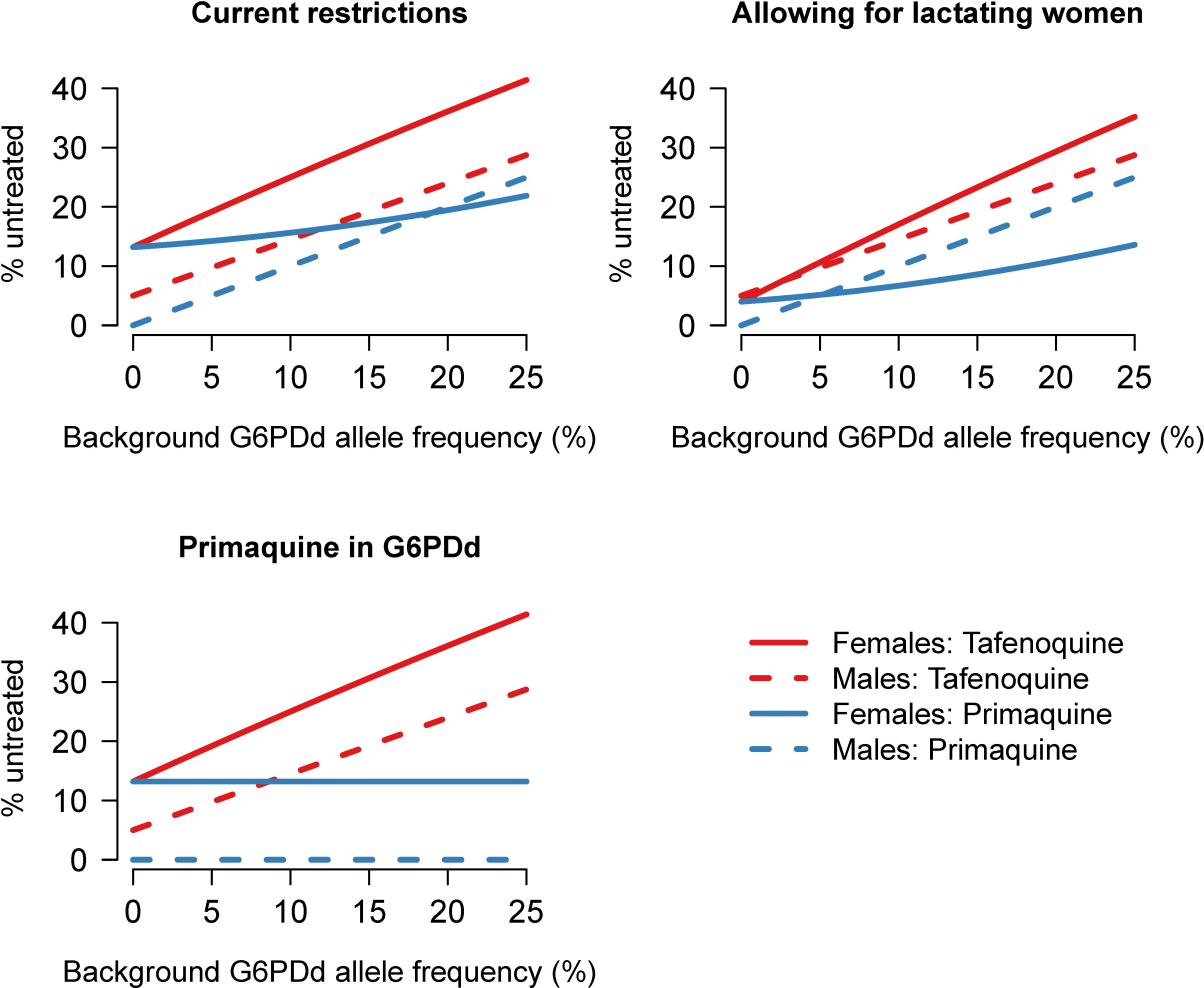
Comparison of prescribing restrictions for primaquine and tafenoquine under three different scenarios broken down by gender. This assumes that 10% of heterozygous females would test deficient at screens with a 30% activity threshold, and 70% of heterozygous females and 5% of all wild type individuals at a 70% threshold. Top left: current proportions of individuals ineligible for radical cure for vivax malaria following G6PD deficiency testing; top right: proportions of individuals ineligible for radical cure if primaquine and tafenoquine could be given to females breast-feeding infants older than 1 month; bottom left: proportions of individuals ineligible for radical cure if primaquine could be given to G6PD deficient persons.

**Fig 3:**
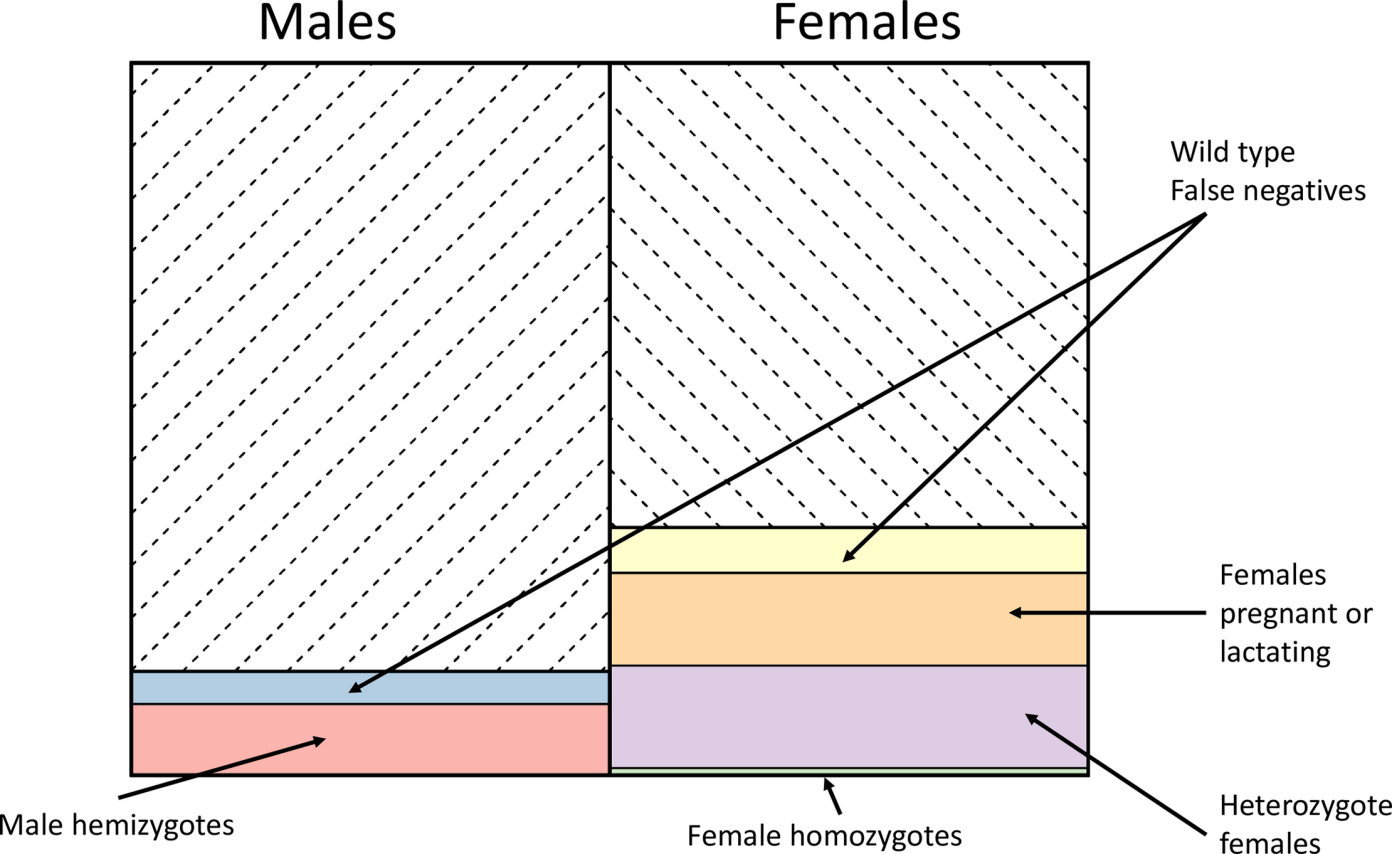
Proportions of individuals who would not be treated with tafenoquine if 10% of males were found to be G6PD deficient. Assumptions as in Fig 2.

Current prescribing restrictions imply that the proportion of males who cannot receive radical cure is the same as the background G6PDd allele frequency. For females, the relationship is more complex due to restrictions in pregnancy and lactation, and G6PD heterozygosity (Fig 2). Under our assumptions, independent of G6PD considerations, pregnancy and lactation result in ~13% of women ineligible for radical cure (6.5% of total population). If the background allele frequency is 10%, then 10 and 14.5% of males cannot receive radical cure (primaquine and tafenoquine, respectively), and 16 and 25% of females cannot receive radical cure (primaquine and tafenoquine, respectively). This results in 13 and 20% of the total population being excluded, respectively. The breakdown of excluded proportions for tafenoquine radical cure with a background allele frequency of 10% is shown in Fig 3.

Sensitivity to these estimates can be computed simply by adjusting the model parameters to fit a variety of epidemiological contexts via an interactive RShiny app found at:

https://moru.shinyapps.io/8_Aminoquinoline_Coverage/.

### Effect of breast-feeding restrictions

Lifting the breast-feeding restrictions for primaquine or tafenoquine would significantly increase potential radical cure coverage. Recent pharmacokinetic studies indicate that very little primaquine is excreted in breast milk, and therefore that primaquine is likely to be safe in breast-feeding mothers with infants older than 28 days [19]. Based on our assumptions [31], 9–10% of women will be breast-feeding at any given time point in the population.

### Alternative primaquine regimen for G6PD deficient individuals

**Fig 4:**
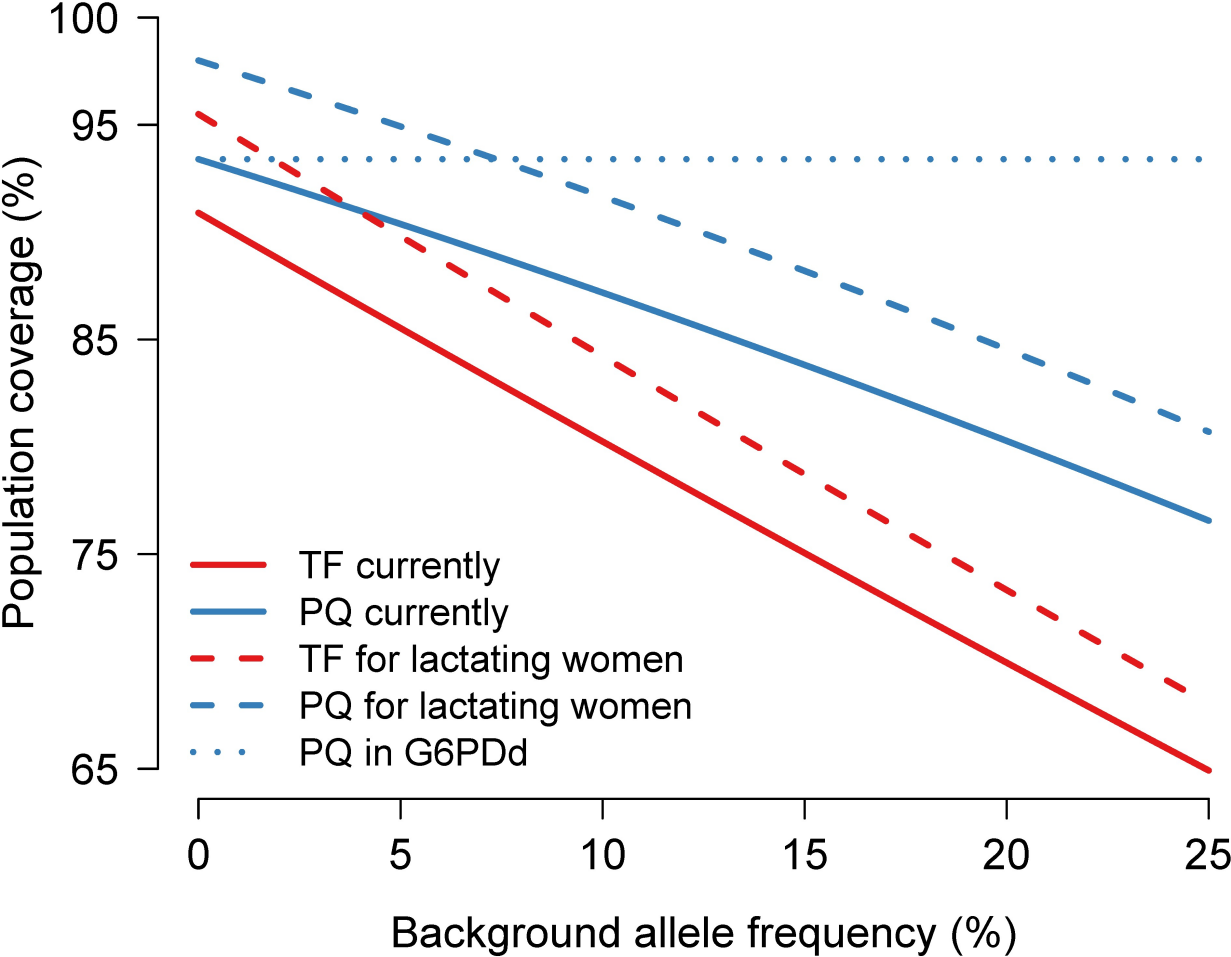
Comparison of estimated treatment coverage for primaquine (blue) and tafenoquine (red) under the three main scenarios considered in this paper. i) current prescribing restrictions (thick lines); ii) 8-aminoquinolines given to G6PD normal lactating women with infants older than 1 month (dashed lines); iii) alternative primaquine regimen for G6PD deficient individuals (dotted line).

If there were an alternative primaquine regimen which could be prescribed safely to G6PDd individuals, this would reduce substantially the proportion of excluded individuals [35] (Fig 2). All males could be treated and only 13% of females (those breast-feeding and pregnant) would be excluded from treatment. This would achieve 93.5% population coverage irrespective of background allele frequency. This compares with 80% coverage for tafenoquine when the background G6PDd allele frequency is 10% (Fig 4).

## Discussion

The majority of patients with vivax malaria who should receive radical curative regimens currently do not receive them. There are many reasons for this including the widespread unavailability of G6PD deficiency testing, concerns over haemolysis in both identified and unidentified G6PD deficiency, restrictions in pregnancy and lactation, the unavailability of primaquine, poor adherence to prescribed regimens and inertia. There is increasing recognition, however, that if vivax malaria is to be controlled and eliminated then the coverage of radical cure does need to increase.

Tafenoquine provides an excellent solution to the problem of poor adherence to standard 14-day primaquine regimens as it is given in a single dose treatment. However, this comes at the price of increased haemolytic risk. Tafenoquine is eliminated slowly and once taken persists at active concentrations in the blood for several weeks (mean half-life 2 weeks) whereas the rapidly eliminated primaquine can be stopped at the first signs of severe AHA. To mitigate the risk of severe AHA in G6PD heterozygous females, most of whom appear G6PD normal on current qualitative RDTs, biosensors have been developed that give a quantitative G6PD enzyme activity result. The percentage of G6PD activity in an individual with reference to the normal population median can be calculated easily. This will allow tafenoquine to be given safely to all males with G6PD activity > 30% and all females with activity > 70% of normal G6PD activity. The field performance of these newly developed sensors has not been assessed yet at scale so it is unknown how many genotypically normal females will be identified as deficient by these tests. However, even if biosensors are made widely available, and they do prove consistently accurate in operational use, substantial proportions of patients will not receive radical cure.

Because of the higher G6PD safety threshold (70%) a greater proportion of individuals, mainly females, will be excluded from tafenoquine compared to daily-administered primaquine (Fig 4). A policy of tafenoquine only in a vivax control programme could mean more than 20% of all individuals would not be eligible to receive radical cure if the G6PDd prevalence is over 10%. This underscores the need to continue provision of alternative safer regimens of primaquine for G6PD deficient patients if tafenoquine is deployed widely. Although once weekly primaquine provides a potential treatment of G6PDd patients, its safety in areas where more severe variants are prevalent has not been well established. The provision of primaquine could be greatly simplified if a regimen were developed that replaced both daily and weekly primaquine and could be used without the need to test for G6PDd [35].

These calculations are illustrative and dependent on assumptions that may not be applicable widely. Population demographics vary widely, mean duration of breast-feeding can often be longer than two years [36], the epidemiology of vivax malaria varies (vivax malaria is mostly a paediatric disease in high transmission areas like New Guinea island), and there is good evidence that severe G6PDd (Mediterranean variant) protects against symptomatic disease [37]. Thus, the proportions of vivax malaria patients excluded from radical cure treatment would be lower than predicted from the allele frequencies. This would be proportional to the severity of the prevalent G6PDd genotypes. In a very recent evaluation using G6PD prevalence data across 95 *P*. *vivax* endemic countries it was estimated that 14.3% of the population would be precluded from primaquine radical cure treatment on safety grounds [12]; in 70% because of G6PD deficiency (in this estimate all heterozygotes were considered excluded), in 12% because of infancy (<6 months), and in 12% because of either pregnancy or lactation (where breast-feeding was for 6 months). Another important consideration is genetic polymorphism in primaquine bioactivation, notably by CYP 2D6. A large number of CYP 2D6 variants have been described which vary from conferring substantial loss of function to gain in function. The *10 variant which confers moderate loss of function is the most common in East Asian populations, reaching an allele prevalence of 43%. Individuals homozygous (or mixed heterozygotes) for loss of function alleles have been reported to have reduced primaquine radical curative efficacy [38,39], and also presumably reduced risk of haemolytic toxicity. Tafenoquine may be less affected by this genetic polymorphism [40].

The current focus of vivax elimination is the administration of radical cure to patients who present with acute disease. However, there is growing evidence that asymptomatic reservoirs of vivax parasitaemia are substantial, most of which are derived from hypnozoites [2,41,42]. If vivax malaria is to be eliminated rapidly, then one approach is to provide focussed mass treatment with 8-aminoquinolines as was done extensively in the past [43]. In that context the protective effect of G6PD deficiency against vivax malaria will not affect these predictions on the proportions of patients who cannot be provided with radical cure.

Tafenoquine may provide substantial operational advantages but it will not obviate the need for primaquine. More work needs to be done to establish the safety or otherwise of alternative primaquine regimens in areas of severe and moderately severe G6PDd variants. High coverage is key to the successful elimination of *Plasmodium vivax*. Tafenoquine will be a significant advance in the management of vivax malaria providing single dose radical cure but a significant proportion of the population (predominantly females) will be unable to receive it. Safer primaquine regimens are needed for these patients.

## Supporting information

S1 Data The extracted meta-data from 9 studies used to estimate the proportion of heterozygous women below the 30 and 70% of population median thresholds, respectively. (XLSX)

S1 Code RMarkdown file providing the R code for the meta-data analysis which produced the estimates given in the paper. (PDF)
