# Supplementary material for: Implications of current therapeutic restrictions for primaquine and tafenoquine in the radical cure of vivax malaria
Source: PLoS Negl Trop Dis. 2018 Apr 20;12(4):e0006440. doi: 10.1371/journal.pntd.0006440 (PMC5931686; doi:10.1371/journal.pntd.0006440)
Supplement: S1 Code — (PDF) [file pntd.0006440.s002.pdf]

Meta-analysis of G6PD activity data

James Watson

Background

This is the statistical analysis presented in the manuscript "Implications of current therapeutic restrictions for primaquine and tafenoquine in the radical cure of vivax malaria", Watson et al. The data are provided as supplementary materials. This R notebook provides a reproducible and transparent analysis.

```
R.version
```

```
##                _
## platform       x86_64-w64-mingw32
## arch           x86_64
## os             mingw32
## system         x86_64, mingw32
## status
## major          3
## minor          4.2
## year           2017
## month          09
## day            28
## svn rev        73368
## language       R
## version.string R version 3.4.2 (2017-09-28)
## nickname       Short Summer
```

Load the data

Read in the first 8 rows of the extracted meta data:

```
rm(list = ls())
meta_data = readxl::read_excel('Extracted_Summary_Data.xlsx')[1:8,]
```

Categorisation of genotypes

In order to partially correct for the over-dispersion in the meta-data, we classify the data into two groups representing levels of severity of G6PD deficient alleles. Our hypothesis is that the deficient allele confers varying average G6PD activities in heterozygous females.

One way of separating out the different levels of severity is by looking at the ratio of hemizygote or homozygous G6PD normals to that of hemizygote or homozygote deficients:

```
dotchart(x = as.numeric(meta_data$`Median value of hemizygotes deficients`) / as.numeric(meta_data$`Norn
     labels = meta_data$`Dominant G6PD mutation(s)`, xlab = 'Ratio of activities: hemizygote deficient /
```

```
## Warning in dotchart(x = as.numeric(meta_data$`Median value of hemizygotes
## deficients`)/as.numeric(meta_data$`Normal Median Value`), : NAs introduced
## by coercion
```

```
## Warning in dotchart(x = as.numeric(meta_data$`Median value of hemizygotes
## deficients`)/as.numeric(meta_data$`Normal Median Value`), : NAs introduced
## by coercion
```

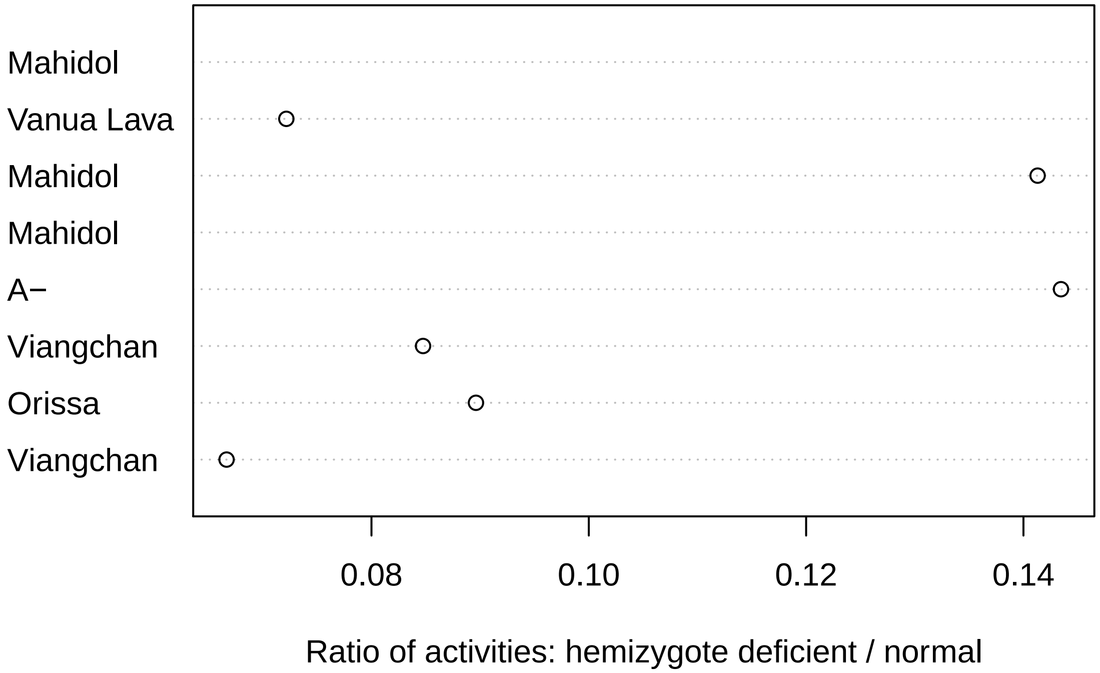

This is only possible for six of the studies due to missing meta-data. We impute for the remaining two studies (both Mahidol).

Bayesian beta-binomial model

We use the *rethinking* package which allows for simple specification of hierachical models which are then fitted using *stan*.

```r
library(rethinking)
```

```
## Loading required package: rstan

## Loading required package: ggplot2

## Loading required package: StanHeaders

## rstan (Version 2.16.2, packaged: 2017-07-03 09:24:58 UTC, GitRev: 2e1f913d3ca3)

## For execution on a local, multicore CPU with excess RAM we recommend calling
## rstan_options(auto_write = TRUE)
## options(mc.cores = parallel::detectCores())

## Loading required package: parallel

## rethinking (Version 1.59)
```

Females heterozygotes below the 30% of population median theshold

Dataframe with columns corresponding to the total sample size for each trial (*Ni*), the number of estimated females below the 30% threshold (*Mi*) and the genotype category.

```
X1 = data.frame(Ni = as.integer(meta_data$`N Females`),
                Mi = as.integer(round(as.numeric(meta_data$`% of Heterozygotes below 30% of population m
                genotype = as.integer(meta_data$`Level of deficiency*`))
```

Specification of the beta binomial model and fitting via *stan*. We choose the following priors:

- The *beta* parameter for each genotype is given a weakly informative prior centered at 0 (this corresponds to 50%)
- The dispersion parameter is given an exponential prior centered at 2 (rate=1/2). Values above 2 correspond to concentrated (non-dispersed) beta-binomial distributions; values less than 2 correspond to dispersed beta-binomials.

Specification of the model using the *map2stan* function from the *rethinking* package, and then model fitting using *stan*.

```
mod1 = map2stan(alist(Mi ~ dbetabinom(Ni,p,theta),
                      logit(p) <-  beta[genotype],
                      beta[genotype] ~ dnorm(0,5),
                      theta ~ dexp(rate = .5)),
                data = X1,
                iter = 5e4, warmup = 2000)
```

```
## In file included from C:/Users/James/Documents/R/win-library/3.4/BH/include/boost/config.hpp:39:0,
##                  from C:/Users/James/Documents/R/win-library/3.4/BH/include/boost/math/tools/config.h
##                  from C:/Users/James/Documents/R/win-library/3.4/StanHeaders/include/stan/math/rev/co
##                  from C:/Users/James/Documents/R/win-library/3.4/StanHeaders/include/stan/math/rev/co
##                  from C:/Users/James/Documents/R/win-library/3.4/StanHeaders/include/stan/math/rev/co
##                  from C:/Users/James/Documents/R/win-library/3.4/StanHeaders/include/stan/math/rev/ma
##                  from C:/Users/James/Documents/R/win-library/3.4/StanHeaders/include/stan/math.hpp:4
##                  from C:/Users/James/Documents/R/win-library/3.4/StanHeaders/include/src/stan/model/m
##                  from file1ac26603c08.cpp:8:
## C:/Users/James/Documents/R/win-library/3.4/BH/include/boost/config/compiler/gcc.hpp:186:0: warning:
##  #  define BOOST_NO_CXX11_RVALUE_REFERENCES
##  ^
## <command-line>:0:0: note: this is the location of the previous definition
## cc1plus.exe: warning: unrecognized command line option "-Wno-ignored-attributes"
##
## SAMPLING FOR MODEL 'Mi ~ dbetabinom(Ni, p, theta)' NOW (CHAIN 1).
##
## Gradient evaluation took 0 seconds
## 1000 transitions using 10 leapfrog steps per transition would take 0 seconds.
## Adjust your expectations accordingly!
##
##
## Iteration:     1 / 50000 [  0%]  (Warmup)
## Iteration:  2001 / 50000 [  4%]  (Sampling)
## Iteration:  7000 / 50000 [ 14%]  (Sampling)
## Iteration: 12000 / 50000 [ 24%]  (Sampling)
## Iteration: 17000 / 50000 [ 34%]  (Sampling)
## Iteration: 22000 / 50000 [ 44%]  (Sampling)
## Iteration: 27000 / 50000 [ 54%]  (Sampling)
```

```
## Iteration: 32000 / 50000 [ 64%]  (Sampling)
## Iteration: 37000 / 50000 [ 74%]  (Sampling)
## Iteration: 42000 / 50000 [ 84%]  (Sampling)
## Iteration: 47000 / 50000 [ 94%]  (Sampling)
## Iteration: 50000 / 50000 [100%]  (Sampling)
##
##  Elapsed Time: 1.103 seconds (Warm-up)
##                24.503 seconds (Sampling)
##                25.606 seconds (Total)
##
##
## SAMPLING FOR MODEL 'Mi ~ dbetabinom(Ni, p, theta)' NOW (CHAIN 1).
##
## Gradient evaluation took 0 seconds
## 1000 transitions using 10 leapfrog steps per transition would take 0 seconds.
## Adjust your expectations accordingly!
##
##
## WARNING: No variance estimation is
##          performed for num_warmup < 20
##
## Iteration: 1 / 1 [100%]  (Sampling)
##
##  Elapsed Time: 0 seconds (Warm-up)
##                0.001 seconds (Sampling)
##                0.001 seconds (Total)

## Warning: There were 1 divergent transitions after warmup. Increasing adapt_delta above 0.8 may help.
## http://mc-stan.org/misc/warnings.html#divergent-transitions-after-warmup

## Warning: Examine the pairs() plot to diagnose sampling problems

## Computing WAIC

## Constructing posterior predictions

## [ 4800 / 48000 ]
[ 9600 / 48000 ]
[ 14400 / 48000 ]
[ 19200 / 48000 ]
[ 24000 / 48000 ]
[ 28800 / 48000 ]
[ 33600 / 48000 ]
[ 38400 / 48000 ]
[ 43200 / 48000 ]
[ 48000 / 48000 ]
```

Look at the two genotype-dependent coefficients determining the value of the beta-binomial posterior. We compute the centered 90% credible intervals.

```
post1 <- extract.samples(mod1)
round(100*quantile( logistic(post1$b[,1]) , c(0.05,0.5,0.95) ))
```

```
##  5% 50% 95%
##   3   8  19
```

```
round(100*quantile( logistic(post1$b[,2]) , c(0.05,0.5,0.95) ))
```

```
##   5% 50% 95%
##    9  19  36
```

Plot of the posterior distribution:

```
plot(precis(mod1,depth=2), bty='n')
```

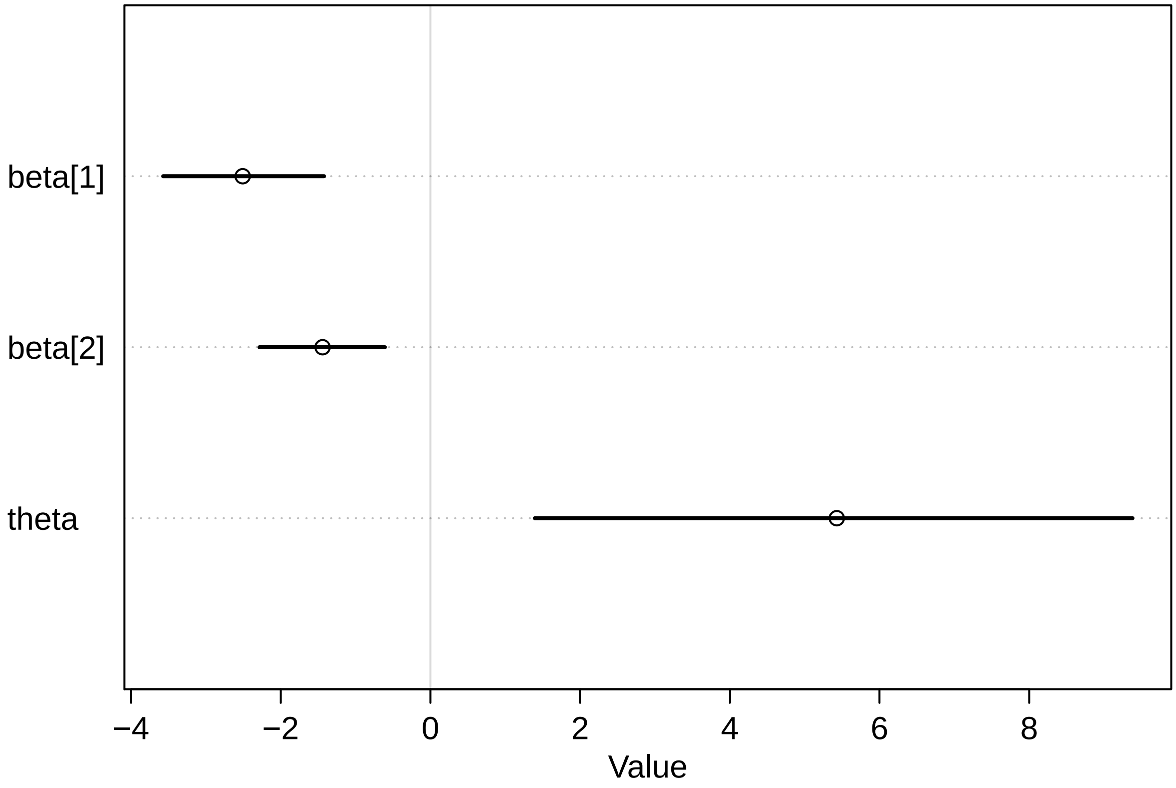

Females heterozygotes below the 70% of population median theshold

Set up dataframe with the estimates of numbers of individuals less than the 70% threshold.

```
X2 = data.frame(Ni = as.integer(meta_data$`N Females`),
                Mi = as.integer(round(as.numeric(meta_data$`% of Heterozygotes below 70% of population m
                genotype = as.integer(meta_data$`Level of deficiency*`))
```

```
## Warning in data.frame(Ni = as.integer(meta_data$`N Females`), Mi =
## as.integer(round(as.numeric(meta_data$`% of Heterozygotes below 70% of
## population median`) * : NAs introduced by coercion
```

```
X2 = X2[!is.na(X2$Mi),]
```

Model specification (same priors as previous section) and model fitting:

```
mod2 = map2stan(alist(Mi ~ dbetabinom(Ni,p,theta),
                      logit(p) <-  beta[genotype],
                      beta[genotype] ~ dnorm(0,5),
                      theta ~ dexp(.5)),
```

```
                 data = X2,
                 iter = 5e4, warmup = 2000)
```

```
## recompiling to avoid crashing R session
```

```
## In file included from C:/Users/James/Documents/R/win-library/3.4/BH/include/boost/config.hpp:39:0,
##                  from C:/Users/James/Documents/R/win-library/3.4/BH/include/boost/math/tools/config.
##                  from C:/Users/James/Documents/R/win-library/3.4/StanHeaders/include/stan/math/rev/c
##                  from C:/Users/James/Documents/R/win-library/3.4/StanHeaders/include/stan/math/rev/c
##                  from C:/Users/James/Documents/R/win-library/3.4/StanHeaders/include/stan/math/rev/c
##                  from C:/Users/James/Documents/R/win-library/3.4/StanHeaders/include/stan/math/rev/m
##                  from C:/Users/James/Documents/R/win-library/3.4/StanHeaders/include/stan/math.hpp:4
##                  from C:/Users/James/Documents/R/win-library/3.4/StanHeaders/include/src/stan/model/
##                  from file1ac30d54a4c.cpp:8:
## C:/Users/James/Documents/R/win-library/3.4/BH/include/boost/config/compiler/gcc.hpp:186:0: warning:
##  #  define BOOST_NO_CXX11_RVALUE_REFERENCES
##  ^
## <command-line>:0:0: note: this is the location of the previous definition
## cc1plus.exe: warning: unrecognized command line option "-Wno-ignored-attributes"
##
## SAMPLING FOR MODEL 'Mi ~ dbetabinom(Ni, p, theta)' NOW (CHAIN 1).
##
## Gradient evaluation took 0 seconds
## 1000 transitions using 10 leapfrog steps per transition would take 0 seconds.
## Adjust your expectations accordingly!
##
##
## Iteration:     1 / 50000 [  0%]  (Warmup)
## Iteration:  2001 / 50000 [  4%]  (Sampling)
## Iteration:  7000 / 50000 [ 14%]  (Sampling)
## Iteration: 12000 / 50000 [ 24%]  (Sampling)
## Iteration: 17000 / 50000 [ 34%]  (Sampling)
## Iteration: 22000 / 50000 [ 44%]  (Sampling)
## Iteration: 27000 / 50000 [ 54%]  (Sampling)
## Iteration: 32000 / 50000 [ 64%]  (Sampling)
## Iteration: 37000 / 50000 [ 74%]  (Sampling)
## Iteration: 42000 / 50000 [ 84%]  (Sampling)
## Iteration: 47000 / 50000 [ 94%]  (Sampling)
## Iteration: 50000 / 50000 [100%]  (Sampling)
##
##  Elapsed Time: 0.878 seconds (Warm-up)
##                19.452 seconds (Sampling)
##                20.33 seconds (Total)
##
##
## SAMPLING FOR MODEL 'Mi ~ dbetabinom(Ni, p, theta)' NOW (CHAIN 1).
##
## Gradient evaluation took 0 seconds
## 1000 transitions using 10 leapfrog steps per transition would take 0 seconds.
## Adjust your expectations accordingly!
##
##
## WARNING: No variance estimation is
##          performed for num_warmup < 20
```

```
##
## Iteration: 1 / 1 [100%]  (Sampling)
##
##  Elapsed Time: 0 seconds (Warm-up)
##                0 seconds (Sampling)
##                0 seconds (Total)
```

```
## Computing WAIC
```

```
## Constructing posterior predictions
```

```
## [ 4800 / 48000 ]
[ 9600 / 48000 ]
[ 14400 / 48000 ]
[ 19200 / 48000 ]
[ 24000 / 48000 ]
[ 28800 / 48000 ]
[ 33600 / 48000 ]
[ 38400 / 48000 ]
[ 43200 / 48000 ]
[ 48000 / 48000 ]
```

Look at the two genotype-dependent coefficients determining the value of the beta-binomial posterior. We compute the centered 90% credible intervals.

```
post2 <- extract.samples(mod2)
round(100*quantile( logistic(post2$b[,1]) , c(0.05,0.5,0.95) ))
```

```
##  5% 50% 95%
##  30  50  70
```

```
round(100*quantile( logistic(post2$b[,2]) , c(0.05,0.5,0.95) ))
```

```
##  5% 50% 95%
##  50  71  86
```

Plot the posterior distributions:

```
plot(precis(mod2,depth=2), bty='n')
```

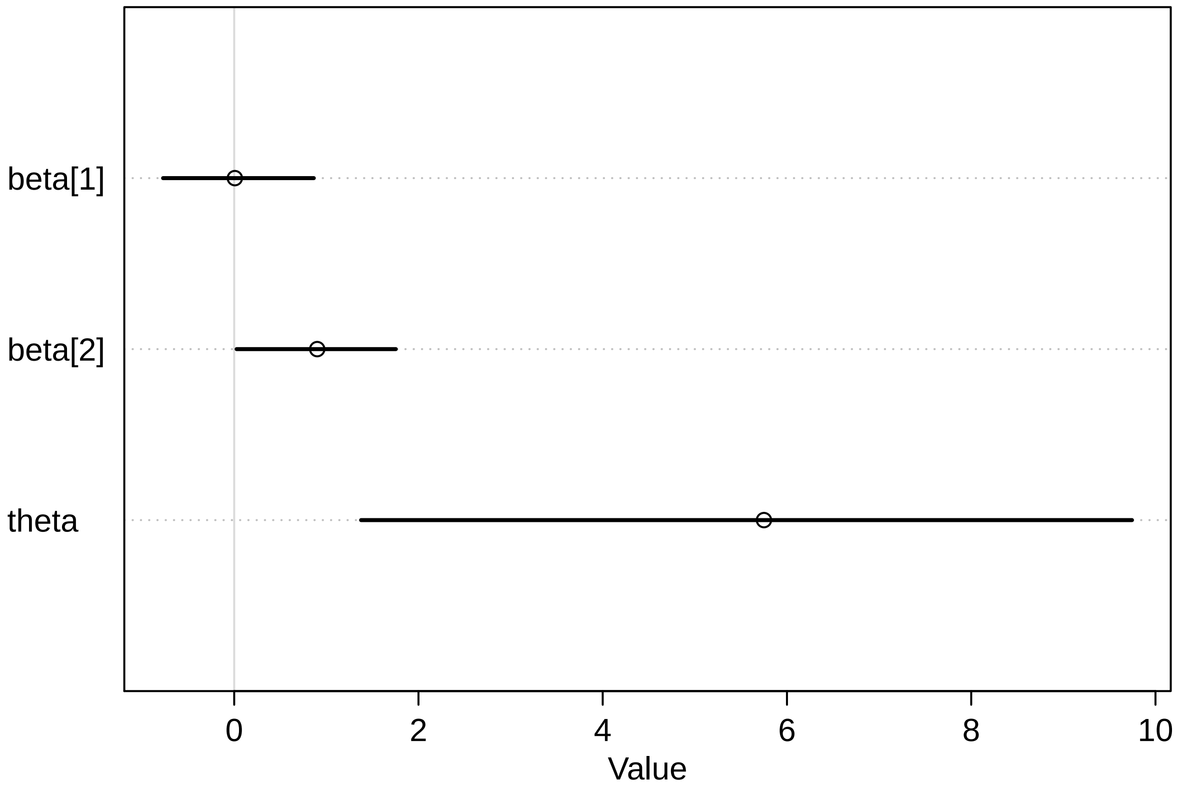
beta[1]
beta[2]
theta
0
2
4
6
8
10
Value
